# A BALANCING ACT: QUALITATIVE DESCRIPTIONS OF DAILY STRESS AMONG BLACK AND WHITE DEMENTIA CAREGIVERS

**DOI:** 10.1093/geroni/igae098.3371

**Published:** 2024-12-31

**Authors:** Angelica Eagle, Emma Weber, Emily Noyer, Angela Turkelson, Kira Birditt

**Affiliations:** University of Michigan, Ann Arbor, Michigan, United States; University of Michigan, Ann Arbor, Michigan, United States; Survey Research Center, Ann Arbor, Michigan, United States; University of Michigan, Ann Arbor, Michigan, United States; University of Michigan, Ann Arbor, Michigan, United States

## Abstract

ADRD caregiving is often stressful and time consuming, having potentially negative implications on the lives of caregivers. This qualitative study described negative daily experiences among ADRD caregivers, considering variations by relationship type and race. Participants from the SWELCare study included 159 ADRD caregivers (97 White, 62 Black) comprising 52 child caregivers, 91 spouse/partner caregivers, and 16 other family or friend caregivers, aged 21 to 86 (M = 62.72, SD = 12.34). Caregivers completed up to 5 interactive voice response interviews for 5 consecutive evenings, providing open-ended responses regarding their most negative event that occurred that day. The data revealed 7 major themes with the most frequently reported regarding: 1) caregiving responsibilities, followed by 2) other daily problems, 3) interpersonal tension with their care recipient, 4) study participation, 5) interpersonal tension with others, 6) physical health problems, and 7) emotional health problems. Over half of the responses included 2 or more themes, indicating negativity across multiple domains. The majority of the descriptions involved issues outside of caregiving tasks. Additionally, White caregivers were significantly more likely to report tension with care recipients than Black caregivers (30% vs 19%). Further, spousal/partner caregivers were significantly more likely to report tension with care recipients than adult child caregivers (31% vs 19%), which suggests interpersonal tension with care recipients vary by race and relationship type. These data identify areas outside of caregiving responsibilities that caregivers may need assistance with, which provides potential targets for improving caregivers’ daily lives.

